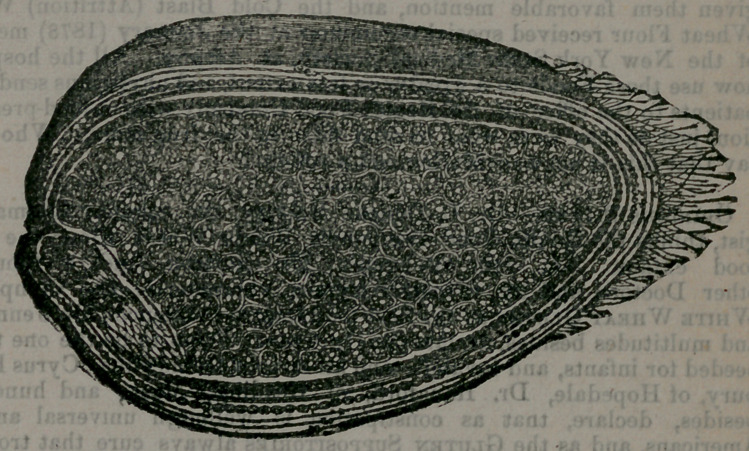# Gluten

**Published:** 1879-08

**Authors:** Frank Fuller


### GLUTEN.

BY FRANK FULLER, A. M., M. D.

Until the HEALTH FOOD COMPANY, of New York, called the attention of the profession and the public to the value of cereal gluten as a nitrogenous aliment, little was known in this country concerning it. In America not an ounce of gluten had ever been completely separated from the starch and bran with which it is associated in nature. It had long been known and used in Europe as a precious and costly food for Diabetics, and a few packages of Connor's Gluten had reached this country from Paris, and had been sold at a dollar a pound. The gluten made by Connor, as well as that made by Van Abbott, in London, has served a useful purpose. since it has furnished an indifferent substitute for bread for those to whom ordinary bread, containing starch, is simply poison; yet the foreign glutens are not only very costly, but are tasteless, insipid, and almost repulsive as foods. The two glutens produced by the Health Food Co. are really palatable foods when skillfully cooked, and the consumer speedily becomes greatly attached to their fine, cleanly, grainy flavor. From a great number of microscopic observations I have made the following drawing for the purpose of showing precisely what gluten is.

Here is a great interior space surrounded with a circle of dark bead-work, which in turn is encircled by five layers or coats. The five layers are the bran-coats, known as epicarp, mesocarp, endocarp, testa, and endosperm. The dark bead-work is the layer of gluten-sacs, and the great interior is the starch-field. Analysis proves that the phosphorous and all other mineral constitutents of the grain—except silex—reside chiefly in this gluten layer. The outer bran-coats are mostly silex and the testa contains a trace of iron; but neither digestion nor chemistry has the power to employ them for practical purposes, although some ignorant patent-medicine vendors have sought to impose upon the credulous by claiming to extract "foods" from these insoluble hulls. The "crude gluten," so-called, is simply the entire gluten sac with its contents; the "purified gluten" is only the contents of the sacs, the walls of cellulose being excluded. The last named gluten resembles the imported in appearance, but is vastly more palatable, and makes a greatly superior bread. The nearest approach to the glutens of this Company was the article called "Cerealine," formerly made by the process of Samuel Bentz, and no longer manufactured. This article contained the testa and the endosperm, as well as considerable starch, this objectionable adjunct being partly removed by sifting and not by water, as practiced by the Health Food Co. It is safe to say that gluten is the best nitrogeneous food known.

## Figures and Tables

**Figure f1:**